# Emergence of Fluoroquinolone-Resistant *Campylobacter jejuni* and *Campylobacter coli* among Australian Chickens in the Absence of Fluoroquinolone Use

**DOI:** 10.1128/AEM.02765-19

**Published:** 2020-04-01

**Authors:** Sam Abraham, Shafi Sahibzada, Kylie Hewson, Tanya Laird, Rebecca Abraham, Anthony Pavic, Alec Truswell, Terence Lee, Mark O’Dea, David Jordan

**Affiliations:** a Antimicrobial Resistance and Infectious Diseases Laboratory, College of Science, Health, Engineering and Education, Murdoch University, Murdoch, Australia; b Australian Chicken Meat Federation, North Sydney, NSW, Australia; c Birling Avian Laboratories, Bringelly, NSW, Australia; d New South Wales Department of Primary Industries, Wollongbar, NSW, Australia; Centers for Disease Control and Prevention

**Keywords:** AMR, Australia, *Campylobacter*, *Campylobacter coli*, *Campylobacter jejuni*, antimicrobial resistance, chicken, fluoroquinolone, genome analysis, livestock

## Abstract

In a structured survey of all major chicken-meat producers in Australia, we investigated the antimicrobial resistance (AMR) and genomic characteristics of Campylobacter jejuni (*n* = 108) and C. coli (*n* = 96) from cecal samples of chickens at slaughter (*n* = 200). The majority of the C. jejuni (63%) and C. coli (86.5%) samples were susceptible to all antimicrobials. Fluoroquinolone resistance was detected among both C. jejuni (14.8%) and C. coli (5.2%), although this only included three sequence types (STs) and one ST, respectively. Multidrug resistance among strains of C. jejuni (0.9%) and C. coli (4.1%) was rare, and fluoroquinolone resistance, when present, was never accompanied by resistance to any other agent. Comparative genome analysis demonstrated that Australian isolates were found dispersed on different branches/clusters within the international collection. The major fluoroquinolone-resistant STs of C. jejuni (ST7323, ST2083, and ST2343) and C. coli (ST860) present in Australian chickens were similar to those of international isolates and have been reported previously in humans and animals overseas. The detection of a subpopulation of *Campylobacter* isolates exclusively resistant to fluoroquinolone was unexpected since most critically important antimicrobials such as fluoroquinolones are excluded from use in Australian livestock. A number of factors, including the low level of resistance to other antimicrobials, the absence of fluoroquinolone use, the adoption of measures for preventing spread of contagion between flocks, and particularly the genomic identities of isolates, all point to humans, pest species, or wild birds as being the most plausible source of organisms. This study also demonstrates the need for vigilance in the form of surveillance for AMR based on robust sampling to manage AMR risks in the food chain.

**IMPORTANCE** *Campylobacter* is one of the most common causes of gastroenteritis in humans, with infections frequently resulting from exposure to undercooked poultry products. Although human illness is typically self-limiting, a minority of cases do require antimicrobial therapy. Ensuring that *Campylobacter* originating from meat chickens does not acquire resistance to fluoroquinolones is therefore a valuable outcome for public health. Australia has never legalized the use of fluoroquinolones in commercial chickens and until now fluoroquinolone-resistant *Campylobacter* has not been detected in the Australian poultry. This structured survey of meat chickens derived from all major Australian producers describes the unexpected emergence of fluoroquinolone resistance in Campylobacter jejuni and C. coli. Genetic characterization suggests that these isolates may have evolved outside the Australian poultry sector and were introduced into poultry by humans, pest species, or wild birds. The findings dramatically underline the critical role of biosecurity in the overall fight against antimicrobial resistance.

## Abstract (precis)

*Campylobacter* is one of the most common causes of gastroenteritis in humans, with infections frequently resulting from exposure to undercooked poultry products. Although human illness is typically self-limiting, a minority of cases do require antimicrobial therapy. Ensuring that *Campylobacter* originating from meat chickens does not acquire resistance to fluoroquinolones is therefore a valuable outcome for public health. Australia has never legalized the use of fluoroquinolones in commercial chickens and until now fluoroquinolone-resistant *Campylobacter* has not been detected in the Australian poultry. This structured survey of meat chickens derived from all major Australian producers describes the unexpected emergence of fluoroquinolone resistance in Campylobacter jejuni and C. coli. Genetic characterization suggests that these isolates may have evolved outside the Australian poultry sector and were introduced into poultry by humans, pest species, or wild birds. The findings dramatically underline the critical role of biosecurity in the overall fight against antimicrobial resistance.

## INTRODUCTION

Campylobacter jejuni and C. coli are common inhabitants of the gastrointestinal tracts of animals and are regarded as the most frequent causes of acute bacterial enteritis in humans (1–4). The main pathways by which humans acquire infection (1, 5) are the consumption of undercooked poultry meat, food cross-contaminated with raw poultry product, water contaminated with *Campylobacter* from animals or humans, and direct contact with animals or human clinical campylobacteriosis cases (4, 6). Campylobacteriosis in humans is usually a self-limiting condition involving diarrhea, abdominal cramping, and fever of up to 2 weeks' duration (1, 2). However, neonates, the elderly, and individuals with immune disorders might develop more serious symptoms that necessitate antimicrobial therapy (2, 7, 8). In such cases, macrolides such as erythromycin are the first choice for treatment, although in many countries the use of ciprofloxacin (a fluoroquinolone) is used preferred on an empirical basis (2). The emergence of resistance to fluoroquinolones in *Campylobacter* spp., combined with the high incidence of *Campylobacter* infections in humans, is a major concern for public health (9).

Many countries have experienced a steady increase in the proportion of *Campylobacter* isolates from humans and animals expressing resistance to fluoroquinolones (10, 11). A recent European Union report on health aspects of antimicrobial resistance (AMR) reported high rates of fluoroquinolone resistance among C. jejuni from broilers (66.9%) and humans (54.6%) (12). A recent review by Sproston et al. (11) also highlighted a global trend in increasing fluoroquinolone resistance among *Campylobacter* isolates from both humans and poultry (11). These events have been attributed to the widespread use of fluoroquinolones in the livestock sector, although in some cases the maintenance of fluoroquinolone-resistant *Campylobacter* strains in livestock has occurred without any direct selection pressure from use of fluoroquinolones (10, 11). A distinctly different circumstance exists in Australia, where the rate of detection of fluoroquinolone resistance in *Campylobacter* spp. from humans is very low (2, 10, 13) and where most such infections are thought to be acquired when traveling abroad (13). The low burden of human infection with fluoroquinolone-resistant *Campylobacter* has also been attributed to the exclusion of this class from registered products available for use in food animals in Australia, as well as the protection provided by geographic isolation and strict quarantine measures at the national border (14, 15).

Although fluoroquinolones are not registered for use in any food-producing animals (including meat chickens) in Australia, it is essential to determine whether there has been sporadic emergence of fluoroquinolone-resistant *Campylobacter*, as well as to identify the frequency of resistance to other antimicrobials. Here, we sought to obtain an epidemiologically sound collection of C. jejuni and C. coli isolates representative of strains harbored by the Australian meat chicken flock and to investigate their antimicrobial resistance and genomic characteristics.

## RESULTS

### Antimicrobial resistance characterization.

**FIG 1:**
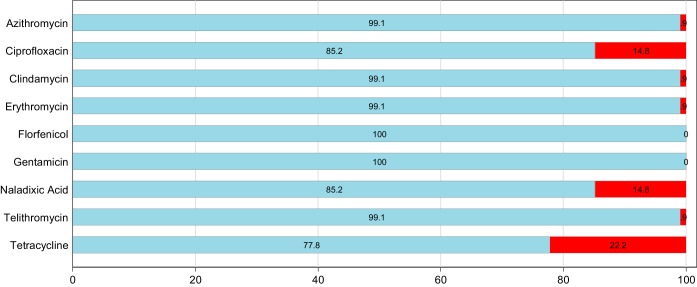
Antimicrobial resistance patterns for C. jejuni (*n* = 108) isolated from Australian meat chickens. The proportion susceptible is shown in blue, and the proportion resistant is shown in red.

**FIG 2:**
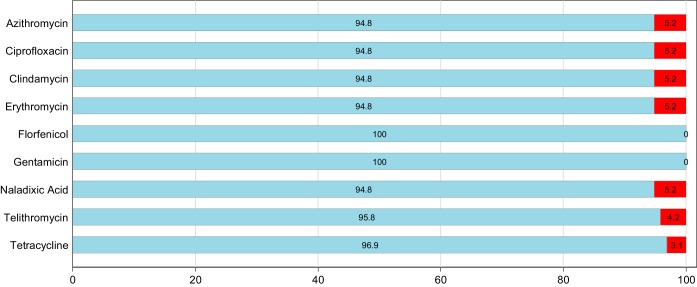
Antimicrobial resistance patterns for C. coli (*n* = 96) isolated from Australian meat chickens. The proportion susceptible is shown in blue, and the proportion resistant is shown in red.

A total of 204 individual isolates of *Campylobacter* (108 C. jejuni isolates and 96 C. coli isolates) were isolated from 200 pooled cecal samples. The antimicrobial resistance patterns for C. jejuni *and* C. coli based on epidemiologic cutoff values (ECOFFs) are shown in Fig. 1 and 2. Full MIC distributions are shown in Tables S2 and S3 in the supplemental material.

### (i) *Campylobacter jejuni*.

Of the 108 C. jejuni isolates, the most commonly detected resistance was to tetracycline (22.2%), followed by resistance to the quinolones ciprofloxacin (14.8%) and nalidixic acid (14.8%) (Fig. 1). In addition, one C. jejuni isolate was also resistant to macrolides (both azithomycin and erythromycin). No resistance was detected to any of the antimicrobials tested in 63% of C. jejuni isolates (Table 1), and none were resistant to florfenicol and gentamicin (Fig. 1). Only one C. jejuni isolate was classified as having a multidrug-resistant (MDR) phenotype (Table 1). The MDR C. jejuni isolate demonstrated resistance to lincosamide, macrolide, and tetracycline.

**TABLE 1:** Class-based antimicrobial resistance profiles of C. jejuni isolates (*n* = 108)

Resistance profile^*a*^	No. of resistances	No. of isolates	% of total
nil	0	68	63.0
qui	1	16	14.8
tet	1	23	21.3
lin_mac_tet	4	1	0.9

a mac, macrolides; nil, no resistance; qui, quinolones; lin, lincosamide; tet, tetracycline.

### (ii) *Campylobacter coli*.

Campylobacter coli isolates displayed less overall resistance to tested antimicrobials, with 86.5% of the 96 isolates susceptable to all tested antimicrobials (Table 2). Commonly detected phenotypic resistance was to ciprofloxacin, nalidixic acid, azithromycin, erythromycin and clindamycin all at 5.2% (Fig. 2). Resistance to telithromycin (4.2%) and tetracylcine (3.1%) was also identified. None of the isolates were resistant to florphenicol or gentamicin.

**TABLE 2:** Class-based antimicrobial resistance profiles of C. coli isolates (*n* = 96)

Resistance profile^*a*^	No. of resistances	No. of isolates	% of total
nil	0	83	86.5
qui	1	5	5.2
tet	1	3	3.1
lin_mac	5	5	5.2

a mac, macrolides; nil, no resistance; qui, quinolones; lin, lincosamide; tet, tetracycline.

### Genomic characterization. (i) *Campylobacter jejuni*.

The C. jejuni isolates demonstrate high genetic diversity, with the isolates belonging to 32 known sequence types and 9 new sequence types. The most prominent sequence types were ST7323 (*n* = 9), ST2083 (*n* = 8), ST535 (*n* = 7), ST4896 (*n* = 7), and 9432 (*n* = 7) (see Table S4 in the supplemental material).

**FIG 3:**
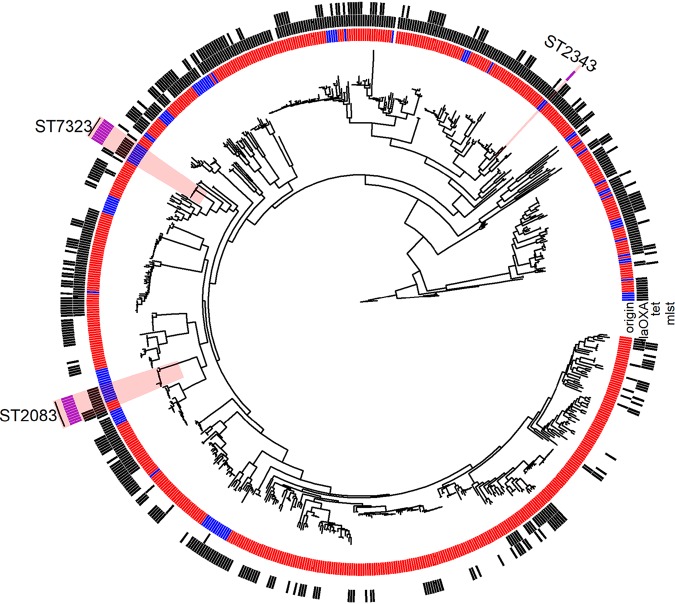
Core genome phylogeny of the 105 Australian (blue) and 628 international collection (red) isolates of C. jejuni. The Australian isolates are dispersed on different branches, along with the international collection. The presence of two resistance genes (*tet* and *bla*_OXA_) found among isolates are indicated as black squares. Moreover, the Australian ciprofloxacin-resistant isolates (ST2083 shown as purple squares) are found on the same node with a similar strain type from the international collection.

When principal component analysis (PCA) based on total gene content was performed on Australian isolates and an international collection of C. jejuni isolates, no distinct clustering was observed for Australian isolates (see Fig. S1 in the supplemental material). Similarly, by using phylogenetic analysis we found that the Australian isolates were dispersed among different branches within the international collection. Moreover, the Australian strains resistant to fluoroquinolone (ST2083) were found on the same node with similar strain types of the international collection (Fig. 3). Genetic analysis indicated that all fluoroquinolone-resistant isolates possessed a mutation in the DNA gyrase A subunit (Thr86→Ile) that was absent from all susceptible isolates. Fluoroquinolone-resistant C. jejuni belonged to sequence types ST7323 (*n* = 9), ST2083 (*n* = 8), and ST2343 (*n* = 1). Minimum spanning tree (MST) analysis demonstrated that the fluoroquinolone-resistant STs are not part of clonal clusters; with ST7323, there were a minimum of four locus variants from its nearest ST, and with ST2082, five locus variants separate (Fig. S2A). Phylogenetic analysis of the fluoroquinolone-resistant C. jejuni isolates revealed low levels of diversity between the STs, with 10 to 90 single nucleotide polymorphism (SNP) differences in the core genome between ST2083 and ST7323 (Fig. S3). Analysis of whole-genome sequencing highlighted a low carriage of resistance genes in C. jejuni isolates, with *tetO* identified in 28.4% (*n* = 23) of the isolates (Fig. 3), supporting the phenotypic resistance to tetracycline; 64.8% (*n* = 68) of the isolates carried the *bla*_OXA_ gene.

### (ii) *Campylobacter coli*.

Of the 96 C. coli isolates, one was a mixed C. jejuni-C. coli culture and was subsequently excluded from whole-genome sequencing. Among the 95 remaining C. coli isolates, the predominant sequence types were ST1181 (*n* = 14), ST827 (*n* = 9), ST3985 (*n* = 9), ST825 (*n* = 8), ST832 (*n* = 7), and ST860 (*n* = 7), with a further five known sequence types identified, as well as 12 new sequence types (Table S5).

**FIG 4:**
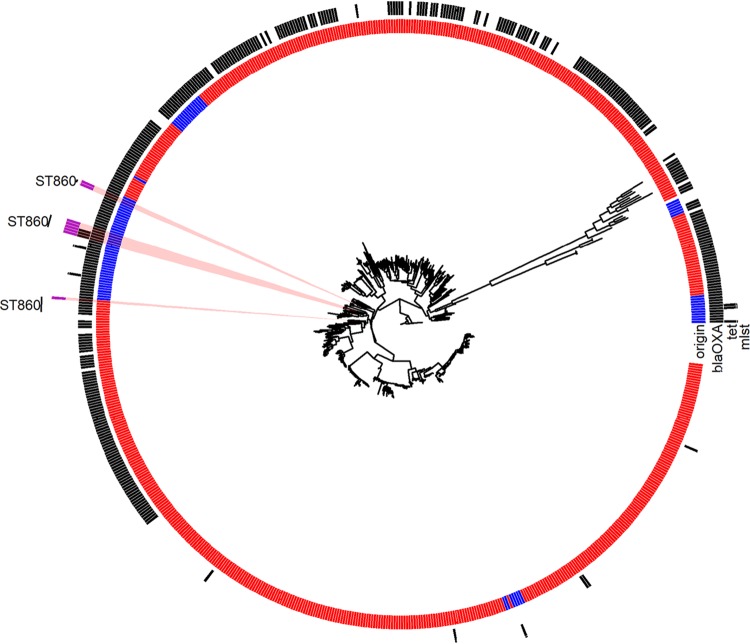
Core genome phylogeny of the 82 Australian (blue) and 647 international collection (red) isolates of C. coli. The Australian isolates are found dispersed on different branches, along with the international collection. The presence of two resistance genes (*tet* and *bla*_OXA_) found among isolates are indicated as black squares. Moreover, the Australian ciprofloxacin-resistant isolates (ST860 shown as purple squares) are found on the same node with a similar strain type from the international collection.

The PCA for total gene content revealed no distinct clustering for Australian isolates compared to the international collection (Fig. S4), a finding corroborated by the phylogenetic analysis of core gene content, where the Australian isolates were dispersed on different branches with the international collection (Fig. 4). The Australian strains resistant to fluoroquinolone (ST860) were found on the same node with similar strain types of the international collection. A separate phylogenetic analysis was also performed for C. coli strains of the Australian collection that resulted in three clades with high levels of diversity between each clade (data not shown), with one clade (Australian clade 1) being particularly divergent from Australian clades 2 and 3 (24,000 and 26,500 SNP differences, respectively). The fluoroquinolone-resistant strains belonged to clade 3. All of the fluoroquinolone-resistant C. coli isolates (5.2%) belonged to ST860; all of these resistant isolates carried the Thr86→Ile mutation in the DNA gyrase subunit associated with fluoroquinolone resistance (Fig. S5). In contrast to fluoroquinolone-resistant C. jejuni isolates, MST analysis appeared to indicate that ST860 may be part of clonal complex linked by single- and double-locus variants involving STs 825, 832, 9417, 9418, 9419, and 3985 (Fig. S2B). There were two isolates belonging to ST860 that had no phenotypic resistance against fluoroquinolone and no mutation for fluoroquinolone resistance (Table S5). Only a single isolate belonging to ST9420 showed a *tet* resistance gene; 72 isolates were positive for the *bla*_OXA_ gene.

Core genome diversity within ST860 revealed fluoroquinolone-susceptible and fluoroquinolone-resistant strains delineated into two major clonal groups. Fluoroquinolone-resistant isolates had core genome SNP differences ranging between 241 and 591 and were between 677 and 736 SNPs different from the susceptible isolates, which were separated by only 214 SNPs (Fig. S5).

## DISCUSSION

In this study, we report the phenotypic antimicrobial resistance and genomic characteristics of C. jejuni and C. coli isolated from a national survey of Australian meat chickens at slaughter, providing an update to surveillance performed in Australian poultry more than 10 years ago (16). Several of our findings stand to progress our understanding of the potential for antimicrobial-resistant C. coli and C. jejuni to adversely impact on humans in Australia. First, we detected low rates of both single-drug antimicrobial resistance and MDR phenotypes, which distinguishes this work from many similar studies performed abroad (10–13). Second, this study reports the detection and genomic characterization of fluoroquinolone-resistant C. jejuni and C. coli in chickens in Australia; these organisms have achieved widespread colonization of commercial flocks without evidence of direct selection pressure from the use of fluoroquinolones. Also, the major STs associated with fluoroquinolone-resistant C. jejuni and C. coli in these Australian isolates from chickens have been reported previously in humans and animals internationally, and the presence of three separate STs in our data set that cluster closely with international strains suggests that at least some of these STs may have been introduced into Australian meat chickens, perhaps through incursions of wild birds or workers returning from overseas. Finally, a high degree of genetic diversity is present in the *Campylobacter* spp. recovered, as indicated by the number of STs present for both C. jejuni (32 STs) and C. coli (10 STs).

Relatively low levels of antimicrobial resistance were observed among both C. jejuni and C. coli strains, with no resistance detected to any of the antibiotics tested in 63% of the C. jejuni isolates and 86.5% of the C. coli isolates. The results for C. jejuni are comparable to the most recent surveillance findings available from Scandinavian countries (17–19); these are jurisdictions noted for their well-established and strict control of antimicrobial use in animals (20). Only 0.9% (1/108) of C. jejuni and 5.2% (5/96) of C. coli isolates were resistant to macrolides (erythromycin and azithromycin), one of the key antimicrobial classes used for treating human campylobacteriosis, and none of the isolates carried an *erm* gene or contained known SNPs in the 23S rRNA genes associated with resistance. The overall frequency of erythromycin resistance among *Campylobacter* spp. in the 2004 survey was 19.9% (16). In the 2004 survey, determination of *Campylobacter* to the species level was not performed (16), but despite this, the current survey shows a substantial reduction in the carriage of macrolide resistance among *Campylobacter* isolates. A low level of MDR phenotypes was identified among both C. coli (4.2%; four isolates) and C. jejuni (0.9%; one isolate) samples, none of which included resistance to fluoroquinolones, but all of which included resistance to macrolides. These outcomes highlight the benefit of the longstanding conservative approach to registration of antimicrobials for use in food animals in Australia.

The four most prominent of the 32 sequence types detected in the C. jejuni isolates from this study have previously been found in humans, with ST2083 and ST535 also found in poultry and ST7323 and ST535 previously reported in Australia (21). The high number of different sequence types detected and the range of hosts and countries where these STs have been detected reveals the high level of diversity in the population of Australian C. jejuni clones. With regard to C. coli, the six main sequence types have all been isolated from humans and livestock previously with ST825, ST827, ST832, ST860, and ST1181 having been reported to cause gastroenteritis in humans. ST825, ST1181, and ST3985 have been isolated from Australian livestock and ST3985 and ST1181 have been isolated from human cases in Australia, with ST832 and ST825 not previously isolated from Australia, as reported to the PubMLST database (21).

The detection of ciprofloxacin resistance in this study was an unexpected finding, given that fluoroquinolones are excluded from use in Australian food animals. Additional evidence that fluoroquinolone resistance did not evolve as a result of local selection pressure is the finding that the responsible mutations are not accompanied by MDR in any isolates. However, the occurrence of fluoroquinolone resistance in the absence of any other resistance phenotype in *Campylobacter* from meat chickens is a phenomenon also observed in other countries with similarly constrained use of fluoroquinolones in food animals (20). For example, ciprofloxacin-resistant C. jejuni was present in 2016 Danish (17) and 2015 U.S. (22) surveillance data at the rates of 23 and 50%, respectively. It is possible that the fluoroquinolone-resistant C. jejuni and C. coli detected in multiple locations globally evolved in wildlife with environmental exposure to fluoroquinolones or from a production system where fluoroquinolones were predominantly used as first-line therapy (23). Recent reports from New Zealand demonstrated that fluoroquinolone resistance detected there among poultry was attributable to the emergence of a new clone of C. jejuni (ST6964) that was resistant to both ciprofloxacin and tetracycline (24). It has been hypothesized that this clone was potentially introduced via exposure to other species (human or other livestock) rather than due to direct antimicrobial use because fluoroquinolones are not registered for use in poultry in New Zealand. Recent Australian studies in livestock have similarly revealed that bacteria expressing resistance to critically important antimicrobials were likely introduced along pathways involving reverse zoonosis (human-animal transmission) or wild birds (20, 25–29). This included the detection of community- and livestock-associated methicillin-resistant Staphylococcus aureus (ST93 and ST398) in pigs (26, 28), community-associated MRSA ST-1 in dairy cows (27), globally disseminated fluoroquinolone and extended-spectrum cephalosporin-resistant E. coli, and plasmids in Australian pigs in the absence of fluoroquinolone use (20, 29).

Importantly, with the exception of horses, the national quarantine boundary of Australia is designed to be impervious to farm animals and unprocessed animal products, thus excluding these pathways as a route of acquisition. *Campylobacter* undergoes significant horizontal transmission, and it has been shown that fluoroquinolone resistance associated with SNPs in the *gyrA* region can be acquired via transformation from resistant isolates (30). However, it would appear that horizontal transfer of genetic material is not the key mechanism involved in resistance, and results from this study, particularly in the case of C. jejuni where fluoroquinolone-resistant isolates appear quite discrete based on MST analysis, would indicate that these STs are separate, and it is reasonable to hypothesize that multiple introductions of clones or episodes of resistance development have occurred. In summary, we postulate that the fluoroquinolone-resistant C. jejuni and C. coli detected in this study were introduced into the Australian chicken industry by mechanisms involving humans and/or wildlife and that this type of transmission might be occurring more commonly than has previously been described. In addition, the role of other incursion pathways, such as contaminated water supplied to the birds for drinking and rodents as reservoirs in farms, also requires further consideration and investigation.

Whole-genome sequence analysis demonstrated that all fluoroquinolone resistant C. jejuni and C. coli isolates possessed the single point mutation (Thr86→Ile) in the DNA gyrase A subunit. Mutations within *gyrA* are associated with fluoroquinolone resistance in multiple bacterial species, with this single nucleotide polymorphism alone conferring fluoroquinolone resistance in *Campylobacter*. The fluoroquinolone-resistant C. jejuni belonged to the ST7323 (*n* = 9), ST2083 (*n* = 8), and ST2343 (*n* = 1) sequence types, which have all been previously isolated from chickens (ST7323 and ST2343 in New Zealand and ST2083 in the United States) (24, 31). These three sequence types have also been isolated from humans (32, 33). Comparative genomic analysis of C. jejuni isolates with international isolates available revealed that fluoroquinolone-resistant C. jejuni isolates belonging to ST2083 were closely related to ST2083 strains isolated from chickens in the United States. The four U.S. C. jejuni isolates belonging to ST2083 all carried the mutation in DNA *gyrA*. However, the lack of genomic data available limits our ability to further investigate the origin of these clones.

ST860 was the only sequence type identified among the fluoroquinolone-resistant C. coli, with this sequence type previously reported in chickens and humans from Vietnam and Japan, respectively (34, 35). With the lack of comprehensive whole-genome sequence and associated source data on epidemiologically relevant sample sizes from different countries that have reported these fluoroquinolone-resistant C. jejuni clones, it is not feasible to perform comparative genome analysis to identify the origins of the fluoroquinolone-resistant isolates identified in this study. Analysis within this sample set demonstrates three fluoroquinolone-resistant C. jejuni clones, which all have low levels of SNP differences within the clones themselves, inferring recent emergence. This is not the case with the C. coli, with the resistant and susceptible strains of ST860 being divergent from one another, suggesting a less recent emergence of the resistant clone.

In conclusion, this study demonstrates a favorable AMR status among the majority of C. jejuni and C. coli isolates with regard to resistance to key antimicrobials important to human health. However, the present study reveals the emergence of fluoroquinolone-specific drug resistance in small subpopulation of C. jejuni and C. coli among Australian isolates from the guts of meat chickens in the absence of fluoroquinolone use. The genomic characterization and phenotypic resistance to fluoroquinolones alone indicates that these isolates may have been introduced to Australian meat chickens via pathways involving reverse zoonosis, pest animals, or wild birds. Follow-up studies to determine circulating *Campylobacter* in wild birds and rodents frequenting production sites and drinking water provided for birds may provide some insight into the origin and ecology of resistant clones.

## MATERIALS AND METHODS

### Study design.

The study was conducted as part of an Australia-wide study (36, 37) based on the collection of composites of five whole cecal pairs from approximately 4- to 7-week-old meat chickens at slaughter between June and November 2016. A total of 200 pooled cecal composites were collected from all major Australian meat chicken producers. The sampling followed a two-stage (cluster based) design involving all major processors of meat chickens in Australia (20 plants representing >95% of production) as the first stage of sampling. Within each plant, the number of cecal composites collected (the second stage of sampling) was proportional to the plant’s processing volume. No more than one cecal composite was obtained from any single processing batch, and each cecal composite was collected immediately postevisceration from midway through the batch (i.e., not the first or last chickens processed from a batch). To construct each composite, only viscera that were not visibly contaminated with digesta were removed with their intact cecal pair, in accordance with the protocol described by NARMS (USA) (38). Each cecal pair was removed from the viscera using sterile scissors at the sphincter between the cecum and the small intestines, and one cecum of each pair was placed into a labeled container (70-ml sterile screw-top containers). This continued until each container held a total of five individual ceca from a single processing batch. Consignments of samples were packed with ice packs and dispatched to the laboratory, where the time elapsed since collection and temperature inside the shipping container were both recorded. Samples that arrived more than 24 h after collection or at a temperature above 8°C were discarded, and a notification was sent to the processing plant for replacement samples to be forwarded.

### Bacterial isolation and identification.

The ceca from each sample were placed into individual stomacher bags and homogenized by stomaching for 60 s and then left standing at room temperature for 5 min for gravity settling of large particles. Campylobacters were isolated using in 90-ml aliquots of *Campylobacter* selective Bolton broth (Thermo Fisher Scientific) by adding 10 g of homogenized sample, followed by shaking to suspend the particles. For samples that were obtained <12 h postsampling, 100 μl was streaked direct from Bolton broth-homogenate onto CSK (Skirrow; bioMérieux) and CFA (Campy Food Agar; bioMérieux) agar and incubated at 42°C for 48 h. For samples that were obtained >12 h postsampling, the direct streaking method was performed, along with a preliminary incubation of the Bolton broth-homogenate sample at 42°C for 48 h under microaerophilic conditions, prior to streaking onto CSK and CFA agar.

Vitek 2 (bioMérieux) was initially used to verify the presence of *Campylobacter* species isolates according to the manufacturer’s instructions. Further confirmation was then performed using MALDI-TOFF (Bruker) by direct plating single colonies onto a matrix-assisted laser desorption ionization–time of flight (MALDI-TOF) target plate, application of alpha-cyano-4-hydroxycinnamic acid matrix solution, and exposure to laser deionization, followed by cross-referencing the output to the Bruker identification library according to the standard manufacturer protocols (Bruker Microflex). From a pure subculture from the original colony, bacteria were harvested for storage at −80°C on proprietarily modified cryo-beads (Thermo Fisher Scientific) until further testing.

### Antimicrobial susceptibility testing.

One cryo-bead from each vial was placed onto a Columbia sheep blood agar (Thermo Fisher Scientific) and incubated microaerophilically at 37°C for 48 h. A single colony was then streaked onto a second Columbia sheep blood agar and incubated at 42°C for 24 h.

Antimicrobial susceptibility for the isolates was determined by the broth microdilution method using the NARMS Campy Sensititre panel (Trek Diagnostics; Thermo Fisher Scientific) according to Clinical and Laboratory Standards Institute guidelines adapted for the Sensititre system (39). The MIC results were captured using the Vision System (Trek; Thermo Fisher Scientific), and results were interpreted and verified independently by two laboratory scientists. The complete list of antimicrobials, along with the concentration ranges that were tested, are listed according to their antimicrobial classes in Table S1 in the supplemental material. Epidemiologic cutoff values (ECOFFs) were used as the basis of interpretation in accordance with the current EUCAST protocol where possible (40). Quality control was performed using C. jejuni ATCC 33560 throughout the study period. In the present study, isolates classified as “non-wild type” based on ECOFF breakpoints are referred to as “resistant.” Isolates resistant to three or more antimicrobial classes were classified as multidrug resistant (MDR).

### Whole-genome sequencing.

A total of 96 C. coli and 108 C. jejuni isolates were selected for whole-genome sequencing. DNA extraction was performed on all isolates using a MagMAX multisample DNA extraction kit (Thermo Fisher Scientific) according to the manufacturer’s instructions. DNA library preparation was conducted by using an Illumina Nextera XT library preparation kit, with variation from the manufacturer’s instructions for an increased time of tagmentation to 7 min. Library preparations were sequenced on an Illumina Nextseq platform using a mid-output 2 × 150 kit. All read data have been deposited in the NCBI database under accession number PRJNA509514. Genomic data were *de novo* assembled using SPAdes (41), and the contigs were analyzed using the Centre for Genomic Epidemiology (http://www.genomicepidemiology.org/) for the screening of multilocus sequence types. Quality checks were performed on all sequenced isolates before analysis of phylogenetic trees and the presence of antimicrobial-resistant and virulence genes. Totals of 82 C. coli and 105 C. jejuni isolates were passed for quality using NASP pipelines using over 80% of quality breadth (42). The antimicrobial genes were searched for and detected using resfinder with a cutoff value of >99% for the identity, with a coverage of 100%. Virulence genes were detected by the Abricate program using the universal virulence finder database with the cutoff of >99% identity and 100% coverage. *Campylobacter* isolates with unknown sequence types were additionally searched against the pubMLST database (C. jejuni*/*C. coli v1.0) (21), with new sequence types assigned. MST results were constructed based on MLST loci using the goeBURST full MST profile within PHYLOViZ (43). The presence of known quinolone resistance region mutations was detected using the Snippy tool (v3.2) in the Nullarbor bioinformatics pipeline (44), and the macrolide-resistant isolates were manually checked for the presence of known resistance associated SNPs at positions 2074 and 2075 of the 23S rRNA gene (45). The *Campylobacter* isolates detected in this study were compared to an international collection of C. jejuni (*n* = 627) and C. coli (*n* = 647) strains previously sequenced in the United States (UDA NARMS data [ENA study accession no. PRJNA292664] and USDA FSIS data [ENA study accession PRJNA287430]) and Spain (46). Phylogenetic trees were constructed from the extraction of all SNPs from the core genome using Snippy and Snippy core (47). ClonalFrameML was used to remove potential recombination (48), and the adjusted core SNP alignment was used to produce a maximum-likelihood tree using RAxML (49). The ggtree package in R was used to annotate phylogenetic trees (50).

### Statistical analysis.

Data were processed using custom scripts for converting plate reader output into MIC tables. Proportions of colonies with traits of interest and the corresponding 95% exact binomial confidence intervals were derived using the Clopper-Pearson method. All analysis was performed using Stata v15.1 (StataCorp LLC, College Station, TX) or the R Statistical Package v3.5.1 (51).

### Data availability.

All read data were deposited in the NCBI database under accession number PRJNA509514.

## Supplementary Material

Supplemental file 1

## References

[1] Wagenaar JA, French NP, Havelaar AH 2013 Preventing *Campylobacter* at the source: why is it so difficult? Clin Infect Dis 57:1600–1606. doi:10.1093/cid/cit555. 24014733

[2] Luangtongkum T, Jeon B, Han J, Plummer P, Logue CM, Zhang Q 2009 Antibiotic resistance in *Campylobacter*: emergence, transmission, and persistence. Future Microbiol 4:189–200. doi:10.2217/17460913.4.2.189. 19257846 PMC2691575

[3] Ruiz-Palacios GM 2007 The health burden of *Campylobacter* infection and the impact of antimicrobial resistance: playing chicken. Clin Infect Dis 44:701–703. doi:10.1086/509936. 17278063

[4] Silva J, Leite D, Fernandes M, Mena C, Gibbs PA, Teixeira P 2011 *Campylobacter* spp. as a foodborne pathogen: a review. Front Microbiol 2:200. doi:10.3389/fmicb.2011.00200. 21991264 PMC3180643

[5] Müllner P, Collins-Emerson JM, Midwinter AC, Carter P, Spencer SE, van der Logt P, Hathaway S, French NP 2010 Molecular epidemiology of *Campylobacter jejuni* in a geographically isolated country with a uniquely structured poultry industry. Appl Environ Microbiol 76:2145–2154. doi:10.1128/AEM.00862-09. 20154115 PMC2849254

[6] Nelson JM, Chiller TM, Powers JH, Angulo FJ 2007 Fluoroquinolone-resistant *Campylobacter* species and the withdrawal of fluoroquinolones from use in poultry: a public health success story. Clin Infect Dis 44:977–980. doi:10.1086/512369. 17342653

[7] Pacanowski J, CAMPYL Study Group, Lalande V, Lacombe K, Boudraa C, Lesprit P, Legrand P, Trystram D, Kassis N, Arlet G, Mainardi J-L, Doucet-Populaire F, Girard P-M, Meynard J-L 2008 *Campylobacter* bacteremia: clinical features and factors associated with fatal outcome. Clin Infect Dis 47:790–796. doi:10.1086/591530. 18699745

[8] Perlman DM, Ampel NM, Schifman RB, Cohn DL, Patton CM, Aguirre ML, Wang WL, Blaser MJ 1988 Persistent *Campylobacter jejuni* infections in patients infected with the human immunodeficiency virus (HIV). Ann Intern Med 108:540–546. doi:10.7326/0003-4819-108-4-540. 3348562

[9] World Health Organization. 2017 Critically important antimicrobials for human medicine: ranking of antimicrobial agents for risk management of antimicrobial resistance due to non-human use. World Health Organization, Geneva, Switzerland.

[10] Price LB, Johnson E, Vailes R, Silbergeld E 2005 Fluoroquinolone-resistant *Campylobacter* isolates from conventional and antibiotic-free chicken products. Environ Health Perspect 113:557–560. doi:10.1289/ehp.7647. 15866763 PMC1257547

[11] Sproston EL, Wimalarathna HML, Sheppard SK 2018 Trends in fluoroquinolone resistance in *Campylobacter*. Microb Genom 4:e000198. doi:10.1099/mgen.0.000198. PMC6159550 30024366

[12] EFSA (European Food Safety Authority) and ECDC (European Centre for Disease Prevention and Control). 2018 The European Union summary report on antimicrobial resistance in zoonotic and indicator bacteria from humans animals and food in 2016. EFSA/ECDC, Parma, Italy https://efsa.onlinelibrary.wiley.com/doi/abs/10.2903/j.efsa.2018.5182. 10.2903/j.efsa.2018.5182 PMC7009656 32625816

[13] Unicomb L, Ferguson J, Riley TV, Collignon P 2003 Fluoroquinolone resistance in *Campylobacter* absent from isolates, Australia. Emerg Infect Dis 9:1482–1483. doi:10.3201/eid0911.030336. 14718099 PMC3035555

[14] Shaban R, Simon G, Trott D, Turnidge J, Jordan D 2014 Surveillance and reporting of antimicrobial resistance and antibiotic usage in animals and agriculture in Australia. Department of Agriculture, The Australian Government, Canberra, Australia.

[15] Unicomb LE, Australian *Campylobacter* Subtyping Study Group, Ferguson J, Stafford RJ, Ashbolt R, Kirk MD, Becker NG, Patel MS, Gilbert GL, Valcanis M, Mickan L 2006 Low-level fluoroquinolone resistance among *Campylobacter jejuni* isolates in Australia. Clin Infect Dis 42:1368–1374. doi:10.1086/503426. 16619147

[16] Australian Department of Agriculture, Fisheries, and Forestry. 2007 Pilot surveillance program for Antimicrobial resistance in bacteria of animal origin. Australian Government Department of Agriculture, Fisheries and Forestry, Canberra, Australia https://www.agriculture.gov.au/animal/health/amr/antimicrobial-resistance-bacteria-animal-origin.

[17] Borck Høg B, Korsgaard HB, Wolff-Sönksen U, Bager F, Bortolaia V, Ellis-Iversen J, Vorobieva V (ed). 2017 DANMAP 2016: use of antimicrobial agents and occurrence of antimicrobial resistance in bacteria from food animals, food and humans in Denmark. Statens Serum Institut/National Veterinary Institute/Technical University of Denmark National Food Institute/Technical University of Denmark, Copenhagen, Denmark.

[18] NORM/NORM-VET. 2016 Usage of antimicrobial agents and occurrence of antimicrobial resistance in Norway. NORM/NORM-VET, Tromsø, Norway.

[19] Swedres-Svarm. 2015 Consumption of antibiotics and occurrence of antibiotic resistance in Sweden. Swedres-Svarm, Solna, Sweden.

[20] Abraham S, Kirkwood RN, Laird T, Saputra S, Mitchell T, Singh M, Linn B, Abraham RJ, Pang S, Gordon DM, Trott DJ, O’Dea M 2018 Dissemination and persistence of extended-spectrum cephalosporin-resistance encoding IncI1-blaCTXM-1 plasmid among *Escherichia coli* in pigs. ISME J 12:2352–2362. doi:10.1038/s41396-018-0200-3. 29899511 PMC6155088

[21] Jolley KA, Bray JE, Maiden MC 2018 Open-access bacterial population genomics: BIGSdb software, the PubMLST.org website and their applications. Wellcome Open Res 3:124. doi:10.12688/wellcomeopenres.14826.1. 30345391 PMC6192448

[22] NARMS. 2017 The National Antimicrobial Resistance Monitoring System: NARMS integrated report, 2015. U.S. Department of Health and Human Services, FDA, Laurel, MD.

[23] Hakanen A, Jousimies-Somer H, Siitonen A, Huovinen P, Kotilainen P 2003 Fluoroquinolone resistance in *Campylobacter jejuni* isolates in travelers returning to Finland: association of ciprofloxacin resistance to travel destination. Emerg Infect Dis 9:267–270. doi:10.3201/eid0902.020227. 12604004 PMC2901943

[24] Muellner P, Kells NJ, Campbell D 2016 The emergence of Campylobacter jejuni ST 6964 in poultry in New Zealand and its associated antimicrobial resistance. Ministry for Primary Industries, Wellington, New Zealand https://www.epi-interactive.com/sites/default/files/documents/2016-16-Risk-Profile-Campylobacter-ST6964-with-SIS.PDF.

[25] Mukerji S, Stegger M, Truswell AV, Laird T, Jordan D, Abraham RJ, Harb A, Barton M, O’Dea M, Abraham S 2019 Resistance to critically important antimicrobials in Australian silver gulls (*Chroicocephalus novaehollandiae*) and evidence of anthropogenic origins. J Antimicrob Chemother 74:2566–2574. doi:10.1093/jac/dkz242. 31287537

[26] Groves MD, O’Sullivan MVN, Brouwers HJM, Chapman TA, Abraham S, Trott DJ, Al Jassim R, Coombs GW, Skov RL, Jordan D 2014 *Staphylococcus aureus* ST398 detected in pigs in Australia. J Antimicrob Chemother 69:1426–1428. doi:10.1093/jac/dkt526. 24446423

[27] Abraham S, Jagoe S, Pang S, Coombs GW, O’Dea M, Kelly J, Khazandi M, Petrovski KR, Trott DJ 2017 Reverse zoonotic transmission of community-associated MRSA ST1-IV to a dairy cow. Int J Antimicrob Agents 50:125–126. doi:10.1016/j.ijantimicag.2017.05.001. 28502696

[28] Sahibzada S, Abraham S, Coombs GW, Pang S, Hernández-Jover M, Jordan D, Heller J 2017 Transmission of highly virulent community-associated MRSA ST93 and livestock-associated MRSA ST398 between humans and pigs in Australia. Sci Rep 7:5273. doi:10.1038/s41598-017-04789-0. 28706213 PMC5509732

[29] Abraham S, Jordan D, Wong H, Johnson J, Toleman M, Wakeham D, Gordon D, Turnidge J, Mollinger J, Gibson J, Trott D 2015 First detection of extended-spectrum cephalosporin- and fluoroquinolone-resistant *Escherichia coli* in Australian food-producing animals. J Glob Antimicrob Resist 3:273–277. doi:10.1016/j.jgar.2015.08.002. 27842872

[30] Jeon B, Muraoka W, Sahin O, Zhang Q 2008 Role of Cj1211 in natural transformation and transfer of antibiotic resistance determinants in *Campylobacter jejuni*. Antimicrob Agents Chemother 52:2699–2708. doi:10.1128/AAC.01607-07. 18505858 PMC2493120

[31] Ladely SR, Berrang ME, Meinersmann RJ, Cox NA 2017 *Campylobacter* multilocus sequence types and antimicrobial susceptibility of broiler cecal isolates: a two-year study of 143 commercial flocks. J Food Safety 37:e12366. doi:10.1111/jfs.12366.

[32] Jolley KA, Maiden MC 2010 BIGSdb: scalable analysis of bacterial genome variation at the population level. BMC Bioinformatics 11:595. doi:10.1186/1471-2105-11-595. 21143983 PMC3004885

[33] Williamson D, Dyet K, Heffernan H 2015 Antimicrobial resistance in human isolates of Campylobacter jejuni, 2015. The Institute of Environmental Science and Research, Ltd, Wellington, New Zealand.

[34] Yabe S, Higuchi W, Iwao Y, Takano T, Razvina O, Reva I, Nishiyama A, Yamamoto T 2010 Molecular typing of *Campylobacter jejuni* and *C. coli* from chickens and patients with gastritis or Guillain-Barré syndrome based on multilocus sequence types and pulsed-field gel electrophoresis patterns. Microbiol Immunol 54:362–367. doi:10.1111/j.1348-0421.2010.00222.x. 20536735

[35] Nguyen TNM, Hotzel H, El-Adawy H, Tran HT, Le MTH, Tomaso H, Neubauer H, Hafez HM 2016 Genotyping and antibiotic resistance of thermophilic *Campylobacter* isolated from chicken and pig meat in Vietnam. Gut Pathog 8:19. doi:10.1186/s13099-016-0100-x. 27175218 PMC4863348

[36] O’Dea M, Sahibzada S, Jordan D, Laird T, Lee T, Hewson K, Pang S, Abraham R, Coombs GW, Harris T 2019 Genomic, antimicrobial resistance and public health insights into *Enterococcus* spp. from Australian chickens. J Clin Microbiol 57:e00319-19. doi:10.1128/JCM.00319-19. 31118269 PMC6663891

[37] Abraham S, O’Dea M, Sahibzada S, Hewson K, Pavic A, Veltman T, Abraham R, Harris T, Trott DJ, Jordan D 2019 *Escherichia coli* and *Salmonella* spp. isolated from Australian meat chickens remain susceptible to critically important antimicrobial agents. PLoS One 14:e0224281. doi:10.1371/journal.pone.0224281. 31644602 PMC6808415

[38] U.S. Food and Drug Administration. 2010 National Antimicrobial Resistance Monitoring System-Enteric Bacteria (NARMS): 2007 executive report. Department of Health and Human Services, US Food and Drug Administration, Rockville, MD.

[39] CLSI. 2015 Performance standards for antimicrobial susceptibility testing; 25th informational supplement. CLSI document M100-S25. Clinical and Laboratory Standards Institute, Wayne, PA.

[40] EUCAST. 2018 Breakpoint tables for interpretation of MICs and zone diameters: version 8.1, valid from 2018-05-15. http://www.eucast.org/fileadmin/src/media/PDFs/EUCAST_files/Breakpoint_tables/v_8.1_Breakpoint_Tables.pdf. Accessed 25 February 2019.

[41] Bankevich A, Nurk S, Antipov D, Gurevich AA, Dvorkin M, Kulikov AS, Lesin VM, Nikolenko SI, Pham S, Prjibelski AD, Pyshkin AV, Sirotkin AV, Vyahhi N, Tesler G, Alekseyev MA, Pevzner PA 2012 SPAdes: a new genome assembly algorithm and its applications to single-cell sequencing. J Comput Biol 19:455–477. doi:10.1089/cmb.2012.0021. 22506599 PMC3342519

[42] Sahl JW, Lemmer D, Travis J, Schupp JM, Gillece JD, Aziz M, Driebe EM, Drees KP, Hicks ND, Williamson CHD, Hepp CM, Smith DE, Roe C, Engelthaler DM, Wagner DM, Keim P 2016 NASP: an accurate, rapid method for the identification of SNPs in WGS datasets that supports flexible input and output formats. Microb Genom 2:e000074. doi:10.1099/mgen.0.000074. 28348869 PMC5320593

[43] Ribeiro-Gonçalves B, Francisco AP, Vaz C, Ramirez M, Carriço JA 2016 PHYLOViZ Online: web-based tool for visualization, phylogenetic inference, analysis and sharing of minimum spanning trees. Nucleic Acids Res 44:W246–W251. doi:10.1093/nar/gkw359. 27131357 PMC4987911

[44] Seemann TG, Bulach DM, Schultz MB, Kwong JC, Howden BP 2015 Nullarbor. Github https://github.com/tseemann/nullarbor. 13 October 2019.

[45] Bolinger H, Kathariou S 2017 The current state of macrolide resistance in *Campylobacter* spp.: trends and impacts of resistance mechanisms. Appl Environ Microbiol 83:e00416-17. doi:10.1128/AEM.00416-17. 28411226 PMC5452823

[46] Ugarte‐Ruiz M, Stabler R, Dominguez L, Porrero M, Wren B, Dorrell N, Gundogdu O 2015 Prevalence of type VI secretion system in Spanish *Campylobacter jejuni* isolates. Zoonoses Public Health 62:497–500. doi:10.1111/zph.12176. 25496466

[47] Seemann T 2015 Snippy: fast bacterial variant calling from NGS reads. Github https://github.com/tseemann/snippy.

[48] Didelot X, Wilson DJ 2015 ClonalFrameML: efficient inference of recombination in whole bacterial genomes. PLoS Comput Biol 11:e1004041. doi:10.1371/journal.pcbi.1004041. 25675341 PMC4326465

[49] Stamatakis A 2014 RAxML version 8: a tool for phylogenetic analysis and post-analysis of large phylogenies. Bioinformatics 30:1312–1313. doi:10.1093/bioinformatics/btu033. 24451623 PMC3998144

[50] Yu G, Smith DK, Zhu H, Guan Y, Lam T 2017 ggtree: an R package for visualization and annotation of phylogenetic trees with their covariates and other associated data. Methods Ecol Evol 8:28–36. doi:10.1111/2041-210X.12628.

[51] R Project for Statistical Computing. 2018 R: a language and environment for statistical computing, v3.5.1. R Foundation for Statistical Computing, Vienna, Austria.

