# Supplementary material for: Emergence of Fluoroquinolone-Resistant Campylobacter jejuni and Campylobacter coli among Australian Chickens in the Absence of Fluoroquinolone Use
Source: Appl Environ Microbiol. 2020 Apr 1;86(8):e02765-19. doi: 10.1128/AEM.02765-19 (PMC7117913; doi:10.1128/AEM.02765-19)
Supplement: Supplemental file 1 [file AEM.02765-19-s0001.pdf]

Supplementary Material

Table S1. Breakpoints used for susceptibility testing of *Campylobacter* species

Class	Agent	Species	Range (mg/L)	ECOFF[a]
**Aminoglycosides**	Gentamicin	All	0.12 - 32	2
**Ketolides**	Telithromycin	All[b]	0.015 - 8	4
**Lincosamide**	Clindamycin	*C. coli*	0.03 - 16	1
		C. jejuni	0.03 - 16	0.5
**Macrolides**	Azithromycin	*C. coli*	0.015 - 64	0.5
		C. jejuni	0.015 - 64	0.25
	Erythromycin	*C. coli*	0.03 - 64	8
		C. jejuni	0.03 - 64	4
**Phenicols**	Florfenicol	All	0.03 - 64	4
**Quinolones**	Ciprofloxacin	All	0.015 - 64	0.5
	Nalidixic acid	All	4 - 64	16
**Tetracyclines**	Tetracycline	*C. coli*	0.06 - 64	2
		C. jejuni	0.06 - 64	1

[a] EUCAST epidemiological cut-off values (ECOFF) (mg/L)

[b] *C. coli* ECOFF derived from National Antimicrobial Resistance Monitoring System (NARMS) programme, Centre for Disease Control and Prevention (CDC)

Table S2. Distribution of minimum inhibitory concentrations for *Campylobacter jejuni* (n=108) isolated from Australian meat chickens.

	Minimum inhibitory concentration (mg/L)														% Resistant
**Antimicrobial**	**0.016**	**0.03**	**0.06**	**0.13**	**0.25**	**0.5**	**1**	**2**	**4**	**8**	**16**	**32**	**64**	**128**	**(95% CI)**
**Azithromycin**	56.5	38.0	4.6	0.0	0.0	0.0	0.0	0.0	0.0	0.0	0.0	0.0	0.0	0.9	0.9 (0.0 – 5.1)
**Ciprofloxacin**	0.0	4.6	44.4	30.6	5.6	0.0	0.0	0.0	0.9	7.4	6.5	0.0	0.0	0.0	14.8 (8.7 – 22.9)
**Clindamycin**	0.0	17.6	43.5	34.3	3.7	0.0	0.0	0.0	0.0	0.9	0.0	0.0	0.0	0.0	0.9 (0.0 – 5.1)
**Erythromycin**	0.0	0.9	9.3	24.1	53.7	11.1	0.0	0.0	0.0	0.0	0.0	0.0	0.0	0.9	0.9 (0.0 – 5.1)
**Florfenicol**	0.0	0.0	0.0	0.9	8.3	50.0	40.7	0.0	0.0	0.0	0.0	0.0	0.0	0.0	0.0 (0.0 – 3.4)
**Gentamicin**	0.0	0.0	0.0	7.4	50.9	41.7	0.0	0.0	0.0	0.0	0.0	0.0	0.0	0.0	0.0 (0.0 – 3.4)
**Nalidixic acid**	0.0	0.0	0.0	0.0	0.0	0.0	0.0	0.0	65.7	19.4	0.0	0.0	2.8	12.0	14.8 (8.7 – 22.9)
**Telithromycin**	0.0	0.0	4.6	13.9	23.1	49.1	8.3	0.0	0.0	0.0	0.9	0.0	0.0	0.0	0.9 (0.0 – 5.1)
**Tetracycline**	0.0	0.0	20.4	33.3	18.5	4.6	0.9	2.8	0.0	0.9	10.2	6.5	0.9	0.9	22.2 (14.7 – 31.2)

% Resistant: Percentage of isolates classified as non-wild type by EUCAST, and corresponding 95% confidence limits.

Table S3. Distribution of minimum inhibitory concentrations for *Campylobacter coli* (n=96) isolated from Australian meat chickens.

Antimicrobial	Minimum inhibitory concentration (mg/L)														% Resistant (95% CI)
	0.016	0.03	0.06	0.13	0.25	0.5	1	2	4	8	16	32	64	128	
**Azithromycin**	7.3	42.7	36.5	8.3	0.0	0.0	0.0	0.0	0.0	0.0	0.0	0.0	0.0	5.2	5.2 (1.7 – 11.7)
**Ciprofloxacin**	0.0	4.2	39.6	42.7	8.3	0.0	0.0	0.0	2.1	3.1	0.0	0.0	0.0	0.0	5.2 (1.7 – 11.7)
**Clindamycin**	0.0	0.0	11.5	49.0	32.3	2.1	0.0	2.1	2.1	1.0	0.0	0.0	0.0	0.0	5.2 (1.7 – 11.7)
**Erythromycin**	0.0	0.0	0.0	9.4	42.7	28.1	10.4	4.2	0.0	0.0	0.0	0.0	0.0	5.2	5.2 (1.7 – 11.7)
**Florfenicol**	0.0	0.0	0.0	0.0	6.3	43.8	45.8	4.2	0.0	0.0	0.0	0.0	0.0	0.0	0.0 (0.0 – 3.8)
**Gentamicin**	0.0	0.0	0.0	4.2	26.0	68.8	1.0	0.0	0.0	0.0	0.0	0.0	0.0	0.0	0.0 (0.0 – 3.8)
**Nalidixic acid**	0.0	0.0	0.0	0.0	0.0	0.0	0.0	0.0	55.2	39.6	0.0	0.0	2.1	3.1	5.2 (1.7 – 11.7)
**Telithromycin**	0.0	0.0	0.0	11.5	24.0	33.3	15.6	7.3	4.2	0.0	4.2	0.0	0.0	0.0	4.2 (1.1 – 10.3)
**Tetracycline**	0.0	0.0	15.6	43.8	29.2	6.3	2.1	0.0	0.0	0.0	2.1	0.0	0.0	1.0	3.1 (0.6 – 8.9)

% Resistant: Percentage of isolates classified as non-wild type by EUCAST, and corresponding 95% confidence limits.

Table S4. MLST and resistance profile of *Campylobacter jejuni* isolates (n=107)

Clonal Complex	MLST	Number of isolates	QRDR mutation	Ciprofloxacin (S/R)
**N/a**	7323	9	yes	R
**N/a**	2083	8	yes	R
**48**	2343	1	yes	R
**460**	535	7		S
**353**	4896	7		S
**N/a**	9432	7		S
**45**	45	5		S
**354**	528	5		S
**48**	48	4		S
**21**	50	4		S
**52**	161	3		S
**257**	257	3		S
**354**	354	3		S
**45**	6722	3		S
**22**	7888	3		S
**N/a**	9422	3		S
**N/a**	449	2		S
**45**	583	2		S
**257**	7572	2		S
**21**	8470	2		S
**N/a**	9425	2		S
**N/a**	9426	2		S
**N/a**	9429	2		S
**42**	42	1		S
**206**	46	1		S
**21**	190	1		S
**45**	233	1		S
**607**	525	1		S
**22**	567	1		S
**N/a**	791	1		S
	996	1		S
**692**	3804	1		S
**692**	4378	1		S
**1332**	6788	1		S
**N/a**	7013	1		S
**443**	7208	1		S
**45**	8559	1		S
**N/a**	9424	1		S
**N/a**	9427	1		S
**N/a**	9428	1		S
**N/a**	9431	1		S

Isolates grouped by MLST with resistant isolates above the line. Gaps represent no presence. S-sensitive; R-resistant. QRDR; quinolone resistance-determining region.

Table S5. MLST and resistance profile of *Campylobacter coli* (n=95)

Clonal Complex	MLST	Number of isolates	QRDR mutation	Ciprofloxacin (S/R)
**828**	860	5	yes	R
**828**	860	2		S
**828**	1181	14		S
**-**	9419	14		S
**828**	827	9		S
**828**	3985	9		S
**828**	825	8		S
**828**	832	6		S
**-**	9420	4		S
**-**	1243	3		S
**-**	1764	2		S
**-**	9418	2		S
**-**	9421	2		S
**-**	9436	2		S
**-**	2534	1		S
**828**	6755	1		S
	8714	1		S
**-**	9417	1		S
**-**	9423	1		S
**-**	9430	1		S
**-**	9433	1		S
**-**	9434	1		S
**-**	9435	1		S
**-**	-	3		S

Isolates grouped by MLST with the resistant isolates above the line. Gaps represent no presence. S-sensitive; R-resistant. QRDR; quinolone resistance-determining region.

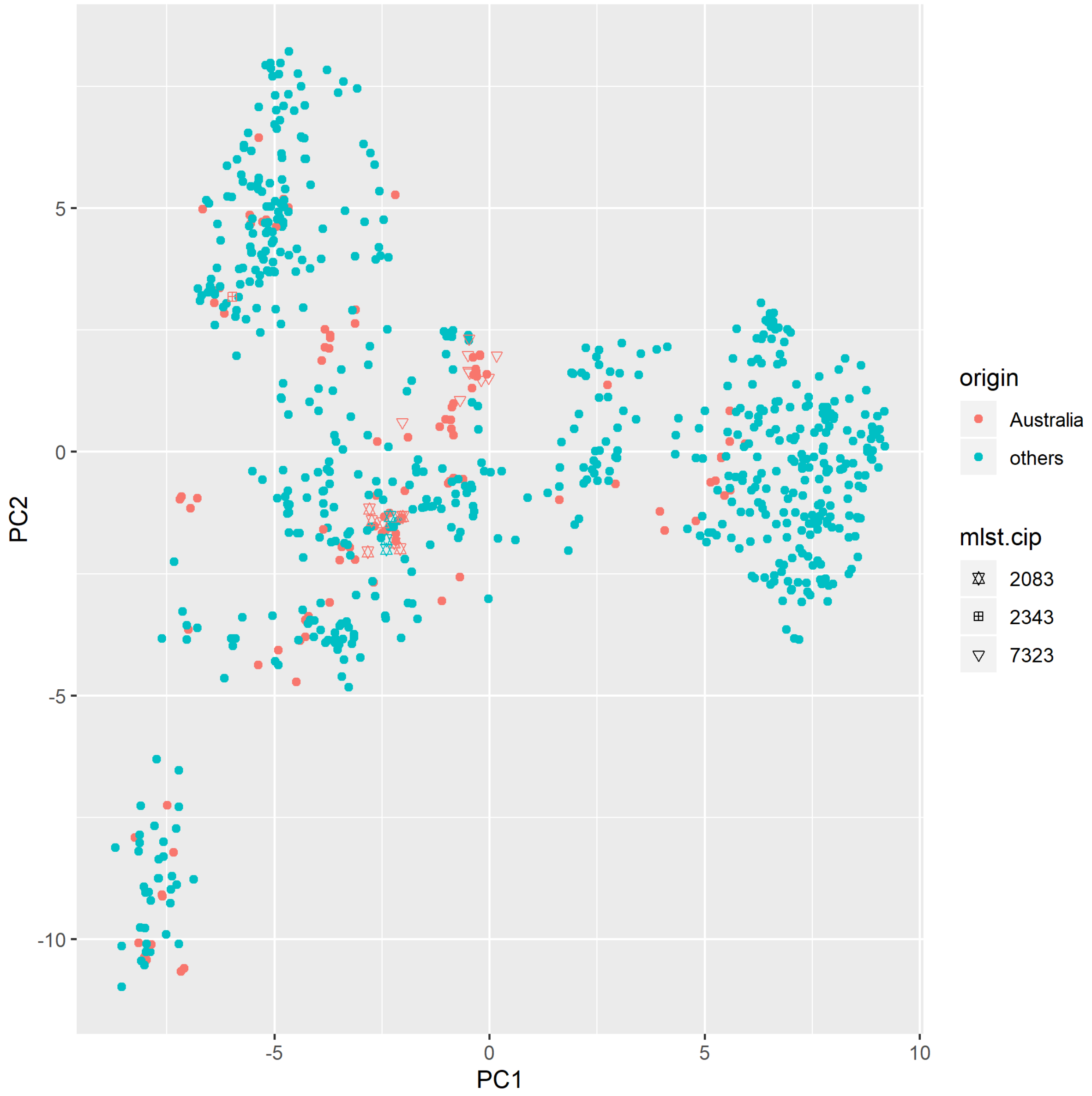

Figure S1. Principal component analysis ordination of total gene content for Australian *C. jejuni* and international collection where no distinct clustering is observed. Strain types resistant to ciprofloxacin are shown as mlst.cip.

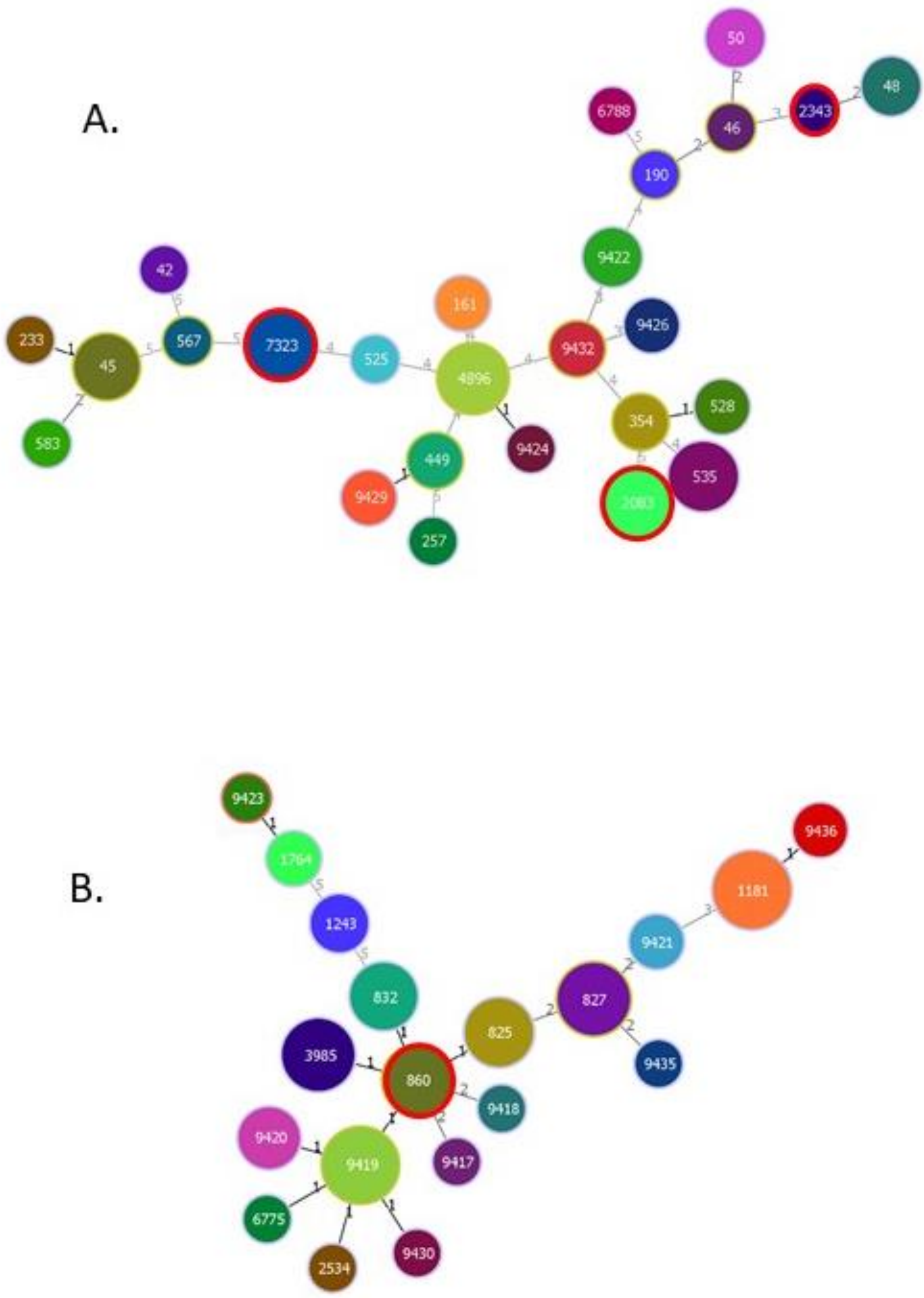

Figure S2. Minimum spanning trees based on MLST loci of *C. jejuni* (A) and *C. coli* (B). Branch numbers indicate number of loci variants, STs in bold red outlines are those with fluoroquinolone resistance.

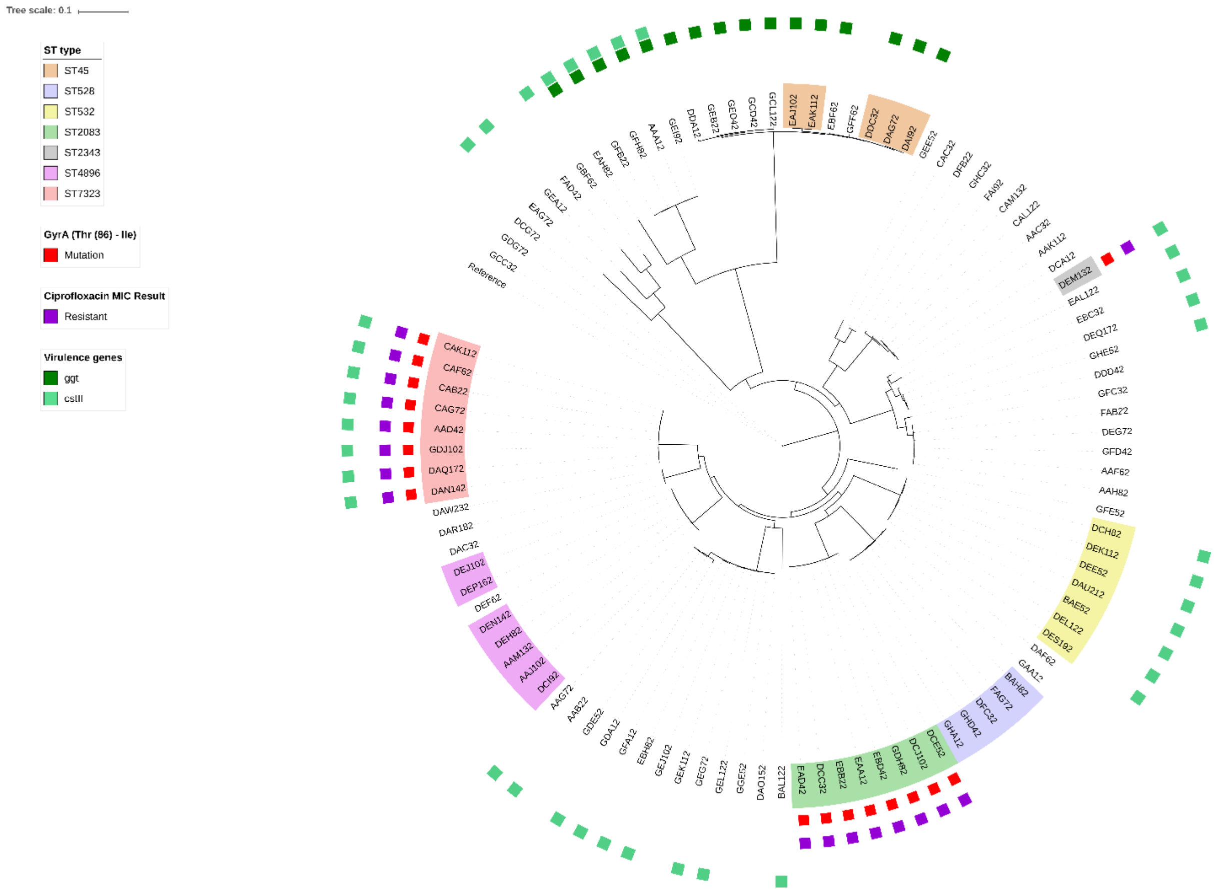

Figure S3. A maximum likelihood phylogeny based on the single nucleotide polymorphisms (SNPs) in the core genome of all *C. jejuni* isolated from Australian chickens. The phylogenetic tree also displays multilocus sequence types, antimicrobial resistance genes, phenotypic and genotypic (mutation of gyrA) resistance to ciprofloxacin of each isolates.

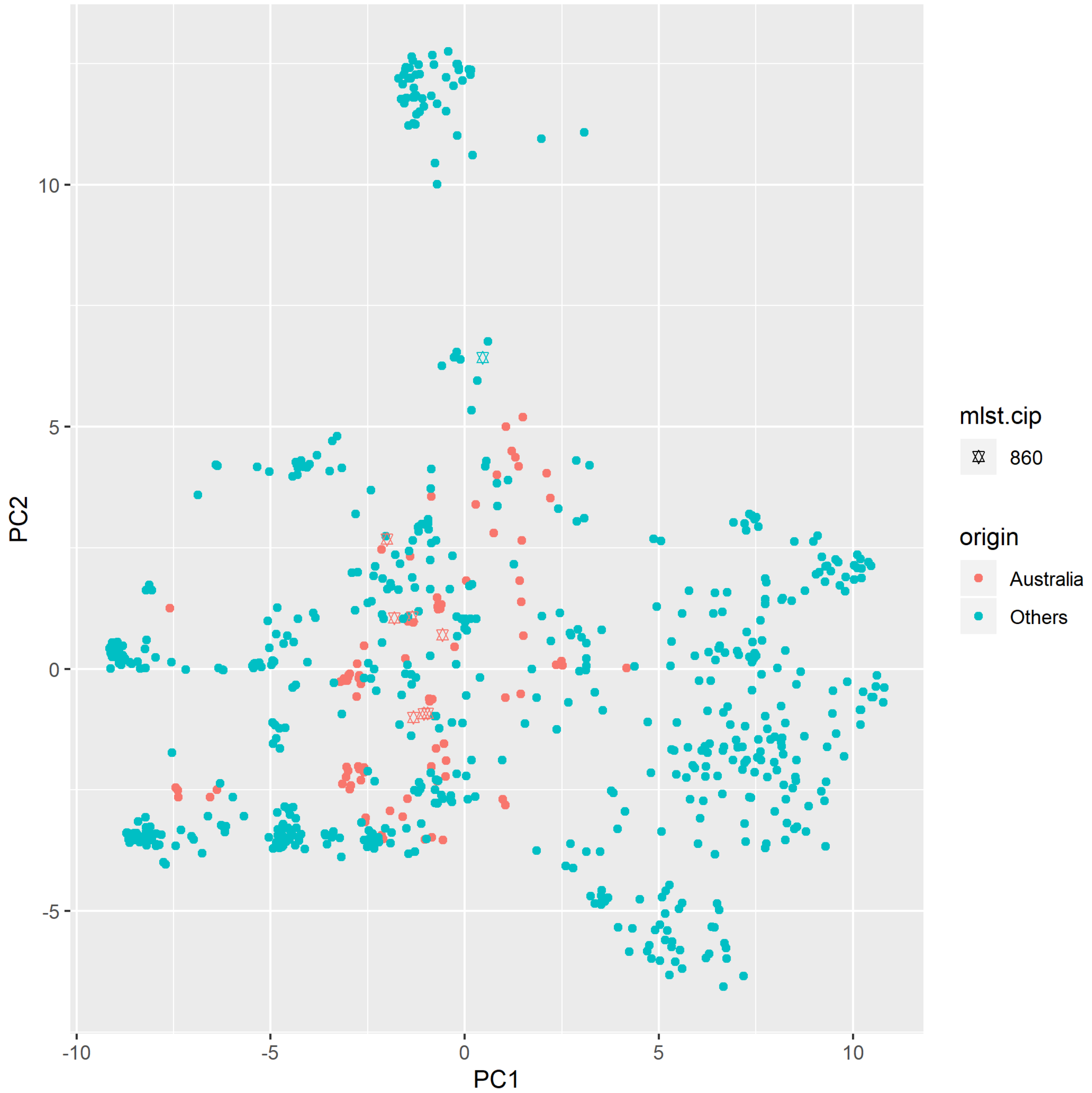

Figure S4. Principal component analysis ordination of total gene content for Australian *C. coli* and international collection where no distinct clustering is observed. Strain types resistant to ciprofloxacin are shown as mlst.cip.

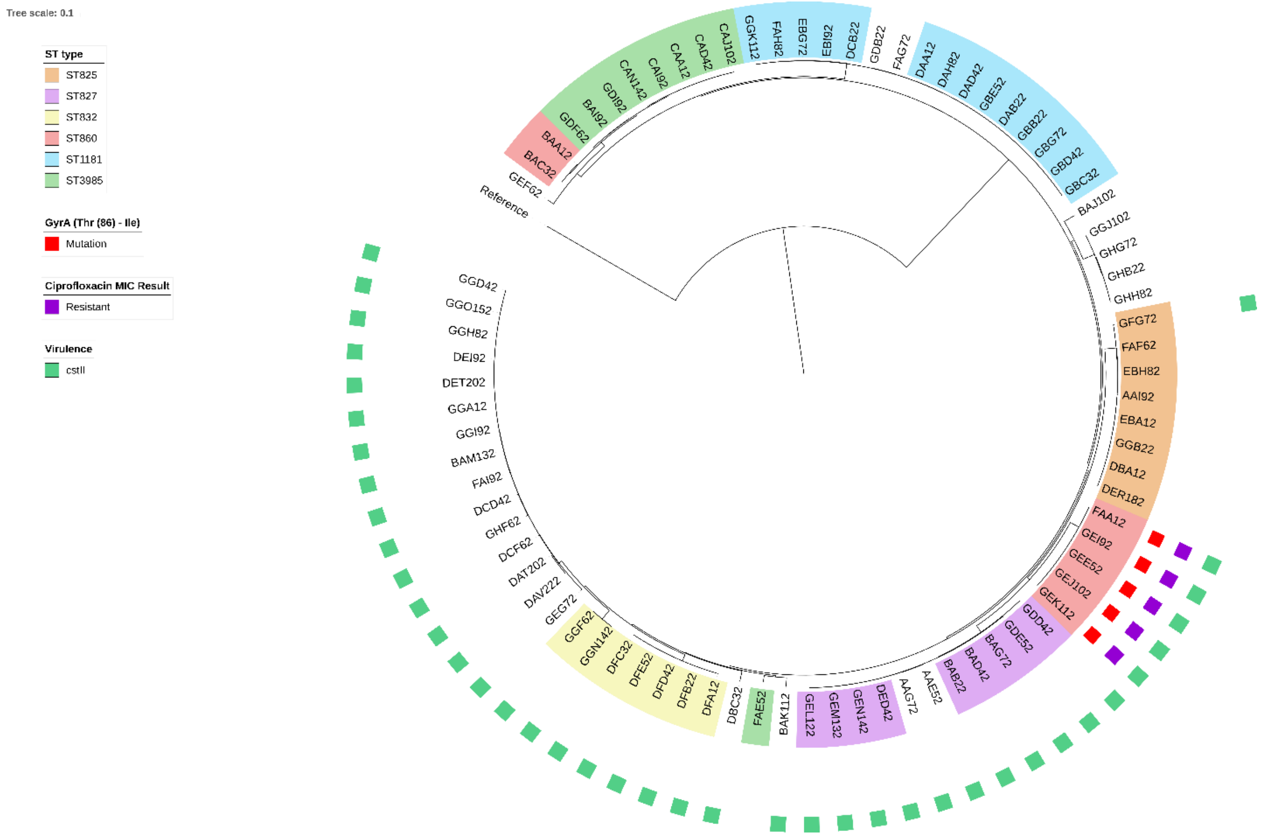

Figure S5. A maximum likelihood phylogeny based on the single nucleotide polymorphisms (SNPs) in the core genome of *C. coli* isolates belonging to Australian clade 3. The phylogenetic tree also displays multilocus sequence types, antimicrobial resistance genes, phenotypic and genotypic (mutation of *gyrA*) resistance to ciprofloxacin of each isolates.
